# Sex and the veins. A cross-sectional analysis on sexual dysfunction in patients with Chronic Venous Disease

**DOI:** 10.1016/j.heliyon.2024.e30038

**Published:** 2024-04-24

**Authors:** Davide Costa, Nicola Ielapi, Umberto Marcello Bracale, Antonio Peluso, Roberto Minici, Michele Andreucci, Raffaele Serra

**Affiliations:** a Department of Law, Economics and Sociology, University “Magna Graecia” of Catanzaro, 88100, Catanzaro, Italy; b Interuniversity Center of Phlebolymphology (CIFL), International Research and Educational Program in Clinical and Experimental Biotechnology. University “Magna Graecia” of Catanzaro, 88100, Catanzaro, Italy; c Department of Public Health and Infectious Disease, Sapienza University of Rome, 00185, Rome, Italy; d Department of Public Health, University of Naples “Federico II”, 80138, Naples, Italy; e Department of Experimental and Clinical Medicine, University “Magna Graecia” of Catanzaro, 88100, Catanzaro, Italy; f Department of Health Sciences. University “Magna Graecia” of Catanzaro, 88100, Catanzaro, Italy; g Department of Medical and Surgical Sciences. University “Magna Graecia” of Catanzaro, 88100, Catanzaro, Italy

**Keywords:** Sexual dysfunction, Chronic venous disease, Quality of life, ASEX

## Abstract

**Background:**

This study aimed to investigate the presence of sexual dysfunction (SD) in patients with Chronic Venous Disease (CVD) and if CVD treatments may have an impact on SD evolution in these patients.

**Methods:**

Inclusion criteria were patients of both sexes and genders, with minimum age of 18, with first diagnosis of CVD. Exclusion criteria were presence of known sexual dysfunction of organic origin, arterial system diseases, malignancies and endocrine system diseases. Included patients were administered the ASEX (Arizona Sexual Experience) questionnaire that was administered at the moment of study inclusion (T0), and for patients that resulted affected from sexual dysfunction, were administered also, after CVD treatments at 6 months (T1) and after 12 months (T2).

**Results:**

A total of 649 patients with CVD were recruited. After the administration of the ASEX questionnaire, 122 patients (18.8 %) resulted affected from SD. Female sex, C3–C6 clinical stages, and the presence of Coronary Artery Disease (CAD), hypertension, and hyperlipidemia were more associated with the presence of SD. SD improved in all patients’ population, especially after CVD treatment at T2.

**Conclusions:**

CVD patients may experience SD, especially in female sex, in more advanced disease stages. SD in CVD patients appears to improve after adequate CVD treatment.

## 1 Introduction

Chronic Venous Disease (CVD) refers to a complex pathophysiology with morphological and functional abnormalities of the superficial venous system that causes severely impaired blood return in the lower limbs. CVD is a widespread clinical condition among general population, especially of the western world, and has important effects on both physical and psychologic aspects of quality of life (QoL) of affected patients [[1], [2], [3], [4], [5]]. In fact, CVD patients have symptoms such as pain, aching, heaviness, feeling of swelling, muscle tiredness, itching, cramps, burning sensations and signs that range from mild manifestations such as telangiectasia, reticular veins, and trunk varicose veins (that are swollen, twisted veins that bulge just under the skin's surface), to more severe ones, such as skin changes (i.e., skin darkening due to hemosiderin deposition, eczematous dermatitis, and lipodermatosclerosis that is a fibrotic evolution of the dermis and subcutaneous fat) and chronic venous ulceration (CVU) of lower limbs. All the aforementioned clinical manifestations are also able to determine important cosmetic dissatisfaction in affected patients [[1], [2], [3], [4], [5], [6]]. Therefore, appropriate management of CVD is mandatory not only for physical issues but also for improving QoL of affected patients, as Booton et al. showed that delaying CVD treatment can even cause significant adverse consequences for QoL including psychological discomfort [7]. Sexual dysfunction (SD) is one of the most common determinants of health affecting also QoL, and as such has both the potential to determine further health problems or, more likely, to be caused by several health issues [8]. SD has been related to cardiovascular diseases, in particular to arterial problems, due to some common pathophysiological pathways, such as endothelial dysfunction and inflammation [9]. To date, no study explored the presence of SD in CVD patients, and the aim of this study is to investigate the possible association of SD in CVD patients and its related characteristics in this population. Also, we aimed to investigate if CVD treatments may influence SD evolution in patients with CVD.

## 2 Materials and methods

This was a cross-sectional clinical study examining consecutive patients of both sex and gender with CVD who were referred to the clinical centers of the University Magna Graecia of Catanzaro and University Federico II of Naples between 2019, January and March 2023. The study was approved by the Institutional Review Board of Interuniversity Center of Phlebolymphology (CIFL) International Research and Educational Program in Clinical and Experimental Biotechnology (Approval number: ER.ALL.2018.64A.). All patients gave written informed consent. The protocol was properly registered at a public trials’ registry, www.clinicaltrial.gov, last accessed on Apr 05, 2023 (NCT05749471). All methods were performed in accordance with the relevant guidelines and regulations.

Inclusion criteria were patients of both sexes, with minimum age of 18, with first diagnosis of CVD. Exclusion criteria were presence of known sexual dysfunction of organic origin, presence of arterial system diseases, presence of malignancies and presence of endocrine system diseases. Each patient was studied according to clinical staging (C) of the clinical, etiological, anatomical, and pathophysiological (CEAP) classification [10].

Included patients were administered the ASEX (Arizona Sexual Experience) questionnaire. ASEX (Arizona Sexual Experience) scale that is considered extremely reliable, valid, and sensitive for measuring sexual dysfunction with optimal internal consistency ASEX is a five-item rating scale that quantifies sex drive, arousal, vaginal lubrication/penile erection, ability to reach orgasm, and satisfaction from orgasm. Possible total scores range from 5 to 30. A subject with a total ASEX score of ≥19, any one item with a score of ≥5, or any three items with a score of ≥4 would have sexual dysfunction [11,12].

The ASEX questionnaire (Table 1) was administered at the moment of study inclusion (T0), and for patients that resulted affected from sexual dysfunction, were administered also, after CVD treatments at 6 months (T1) and after 12 months (T2). CVD treatments in our population study included at least one or a combination of the following, according to CEAP clinical stage, physician's and patient's preferences: veno-active drugs (VADs), open surgery (removal or ligation of varicose veins), endovascular surgery (radiofrequency ablation of great saphenous vein, sclerotherapy), compression therapy (elastic stockings, elastic bandages), and local wound care treatment (wound bed management, wound dressing, etc) in case of chronic venous leg ulcers (CVLUs).

**Table 1:** ASEX questionnaire.

Questionnaire For each item, please indicate your overall level during the past week, including today
**1. How strong is your sex drive?***(rate 1–6)* 1 extremely strong; 2 very strong; 3 somewhat strong; 4 somewhat weak; 5 very weak; 6 no sex drive
**2. How easily are you sexually aroused (turned on)?** *(rate 1–6)* 1 extremely easily; 2 very easily; 3 somewhat easily; 4 somewhat difficult; 5 very difficult; 6 never aroused
**3. Can you easily get and keep an erection** (question for males) *(rate 1–6)* 1 extremely easily; 2 very easily; 3 somewhat easily; 4 somewhat difficult; 5 very difficult; 6 never **How easily does your vagina become moist or wet during sex? (**question for females) *(rate 1–6)* 1 extremely easily; 2 very easily; 3 somewhat easily; 4 somewhat difficult; 5 very difficult; 6 never
**4. How easily can you reach an orgasm?***(rate 1–6)* 1 extremely easily; 2 very easily; 3 somewhat easily; 4 somewhat difficult; 5 very difficult; 6 never reach orgasm
**5. Are your orgasms satisfying?***(rate 1–6)* 1 extremely satisfying; 2 very satisfying; 3 somewhat satisfying; 4 somewhat unsatisfying; 5 very unsatisfying; 6 can't reach orgasm

Statistical analysis was carried out with version April 1, 1106 2009–2021 RStudio, PBC. Continuous subject clinical variables (age, BMI) were analyzed with a Welch two sample *t*-test while categorical variables (females, smoke, hypertension, diabetes, CAD, prior stroke, COPD, CKD, use of drugs, CEAP class and treatment) were considered with a sample test for equality of proportions without continuity correction.

ASEX total score and each of its items have been subjected to Kruskal-Wallis rank-sum test to evaluate possible differences after treatment for CVD.

Improvement quantification of ASEX scores in the subgroup affected by SD, 12 months from CVD treatment (T2), was evaluated with Fisher's test and Pearson's correlation.

## 3 Results

A total of 649 patients with CVD were recruited. After the administration of the ASEX questionnaire, 122 patients (18.8 %) (42 males and 80 females according to biological sex) resulted affected from SD. Twenty-seven patients were lost to follow up. Of these, only 5 patients completed the first follow up visit, and 22 patients did not complete any of the follow up visits.

Therefore, 622 patients completed all the follow up visits. Patients lost to follow-up were not included in statistical analysis.

Demographics are reported in Table 2.

**Table 2:** Subject clinical characteristics.

	Overall (N = 649, 100 %)	CVD (n = 527, 81.2 %)	CVD +SD (n = 122, 18.8 %)	p-value (<0.05)
Subject clinical characteristics				
Age (years)	45 ± 12.6	44.9 ± 12.6	45.3 ± 12.9	0.730
Females	461 (71 %)	381 (72.3 %)	80 (65.5 %)	0.818
BMI (Kg/cm^2^)	25.6 ± 4.2	25.6 ± 4.3	25.5 ± 3.4	0.944
Smoke	219 (33.7 %)	177 (33.5 %)	42 (34.4 %)	0.943
Hypertension (SAP>140 mmHg and/or DAP>90 mmHg)	36 (5.5 %)	23 (4.3 %)	13 (10.6 %)	0.011
Diabetes (glycemia>125 mg/dL)	72 (11.1 %)	57 (10.8 %)	15 (12.3 %)	0.757
Dyslipidemia (Tot. Chol.>240 mg/dL and/or TGL>150 mg/dL)	35 (5.4 %)	23 (4.3 %)	12 (9.8 %)	0.028
CAD	5 (0.7 %)	1 (0.1 %)	4 (3.2 %)	0.003
Prior stroke	2 (0.3 %)	2 (0.3 %)	0 (0 %)	1.000
COPD	6 (0.9 %)	3 (0.5 %)	3 (2.4 %)	0.149
CKD (GFR<60 ml/min/1,73m^2^)	6 (0.9 %)	4 (0.7 %)	2 (1.6 %)	0.696
Use of drugs				
Aspirin	18 (2.7 %)	8 (1.5 %)	10 (8.1 %)	<0.001
DOAC	7 (1 %)	3 (0.5 %)	4 (3.2 %)	0.033
Statin	35 (5.4 %)	23 (4.3 %)	12 (9.8 %)	0.028
Antihypertensive	36 (5.5 %)	23 (4.3 %)	13 (10.6 %)	0.011
CEAP class				
C1	7 (1 %)	7 (1.3 %)	0 (0 %)	0.427
C2	575 (88.6 %)	512 (97.1 %)	63 (51.6 %)	<0.001
C3	36 (5.5 %)	6 (1.1 %)	30 (24.6 %)	<0.001
C4a	9 (1.3 %)	2 (0.3 %)	7 (5.7 %)	<0.001
C4b	12 (1.8 %)	0 (0 %)	12 (9.8 %)	<0.001
C6	10 (1.5 %)	0 (0 %)	10 (8.2 %)	<0.001
Treatment				
EVTA	213 (32.8 %)	157 (29.8 %)	56 (45.9 %)	<0.001
Open surgery	501 (77.2 %)	418 (79.3 %)	83 (68 %)	0.010
Wound care	9 (1.3 %)	0 (0 %)	9 (7.3 %)	<0.001
VADs	536 (82.5 %)	422 (80 %)	114 (93.4 %)	<0.001
CT	569 (87.6 %)	464 (88 %)	105 (86 %)	0.655

*Footnotes:* CVD = chronic venous disease; SD = sexual dysfunction; BMI = body mass index; SAP = systolic arterial blood pressure; DAP = diastolic arterial blood pressure; CAD = coronary artery disease; COPD = chronic obstructive pulmonary disease; CKD = chronic kidney failure; DOAC = direct oral anticoagulant; EVTA = endovenous thermal ablation; VADs = venoactive drugs.

Most subject clinical characteristics were similarly represented in both CVD and CVD +SD groups, except for hypertension, dyslipidemia, and coronary heart disease (CAD) which were more frequent in the CVD +SD group (hypertension: 4.3 % vs 10.6 %, p-value = 0.011; dyslipidemia: 4.3 % vs 9.8 %, p-value = 0.028; CAD: 0.1 % vs 3.2 %, p-value = 0.003).

The rate of patients with clinical CEAP class from C3 to C6, was significantly higher in the group affected by CVD +SD (C3: 1.1 % vs 24.6 %; C4a: 0.3 % vs 5.7 %; C4b: 0 % vs 9.8 %; C6: 0 % vs 8.2 %, p-value= <0.001). Otherwise, CEAP C2 patients were more frequent in the group with CVD alone (C2: 97.1 % vs 51.6 %, p-value= <0.001).

Regarding the CVD treatment options, patients with CDV +SD were more suitable for endovenous thermal ablation (EVTA) than CVD group (EVTA: 29.8 % vs 45.9 %, p-value= <0.001).

After 12 months of CVD treatment (from T0 to T2), the mean of total ASEX score significantly improved (ASEX total score: 21.6 ± 2.9 vs 17.3 ± 2.1, p-value= <0.001). Such improvement was maintained regardless of the sex and the type of CVD treatment employed (Table 3).

**Table 3:** Analysis of ASEX scores before (T0) and after (T1, T2) CVD treatment in the subgroup affected by SD.

CVD +SD *(n = 95*, *14.6 %)*^a^				
	T0 *(mean ± sd)*	T1 *(mean ± sd)*	T2 *(mean ± sd)*	p-value *(<0.05)*
**ASEX total score***(5–30)*				
Overall	21.6 ± 2.9	18.7 ± 2.7	17.3 ± 2.1	<0.001
Males	21.4 ± 2.8	18.6 ± 2.4	17.4 ± 2.1	<0.001
Females	21.7 ± 2.9	18.8 ± 2.9	17.2 ± 2.1	<0.001
EVTA	21.5 ± 2.8	18.4 ± 2.5	17.2 ± 2.1	<0.001
Open surgery	21.8 ± 3	19.1 ± 2.8	17.5 ± 2.1	<0.001
**6. How strong is your sex drive?***(1–6)*				
Overall	4.6 ± 0.7	3.4 ± 0.8	3.1 ± 0.6	<0.001
Males	4.6 ± 0.8	3.4 ± 0.7	3.2 ± 0.5	<0.001
Females	4.6 ± 0.7	3.4 ± 0.8	3 ± 0.6	<0.001
EVTA	4.7 ± 0.7	3.4 ± 0.7	3 ± 0.5	<0.001
Open surgery	4.7 ± 0.8	3.5 ± 0.7	3.1 ± 0.6	<0.001
**7. How easily are you sexually aroused?***(1–6)*				
Overall	4.4 ± 0.8	3.5 ± 0.7	3.2 ± 0.6	<0.001
Males	4.2 ± 0.8	3.4 ± 0.6	3.2 ± 0.5	<0.001
Females	4.5 ± 0.7	3.5 ± 0.7	3.2 ± 0.6	<0.001
EVTA	4.4 ± 0.8	3.4 ± 0.7	3.2 ± 0.6	<0.001
Open surgery	4.4 ± 0.8	3.6 ± 0.7	3.3 ± 0.6	<0.001
**8. Can you easily get and keep an erection/How easily does your vagina become moist or wet during sex?***(1–6)*				
Overall	4 ± 0.8	3.7 ± 0.7	3.3 ± 0.6	<0.001
Males	4 ± 0.8	3.6 ± 0.8	3.3 ± 0.6	<0.001
Females	4.1 ± 0.8	3.7 ± 0.7	3.3 ± 0.5	<0.001
EVTA	4 ± 0.8	3.6 ± 0.7	3.4 ± 0.6	<0.001
Open surgery	4.1 ± 0.8	3.7 ± 0.7	3.4 ± 0.6	<0.001
**9. How easily can you reach an orgasm?***(1–6)*				
Overall	4.2 ± 0.7	4 ± 0.6	3.7 ± 0.5	<0.001
Males	4.2 ± 0.6	4 ± 0.6	3.7 ± 0.5	0.001
Females	4.2 ± 0.7	4 ± 0.6	3.6 ± 0.5	<0.001
EVTA	4.2 ± 0.6	3.9 ± 0.5	3.7 ± 0.6	<0.001
Open surgery	4.2 ± 0.7	4.2 ± 0.7	3.7 ± 0.5	<0.001
**10. Are your orgasms satisfying?***(1–6)*				
Overall	4.2 ± 0.6	4 ± 0.6	3.8 ± 0.6	<0.001
Males	4.2 ± 0.6	4 ± 0.6	3.8 ± 0.6	0.004
Females	4.2 ± 0.7	4.1 ± 0.7	3.9 ± 0.5	0.002
EVTA	4.2 ± 0.6	4.2 ± 0.6	3.8 ± 0.6	0.005
Open surgery	4.3 ± 0.7	4.1 ± 0.7	3.8 ± 0.6	0.001

*Footnotes:* CVD = chronic venous disease; SD = sexual dysfunction; T0 = time of CVD diagnosis; T1 = 6 months after CVD treatment; T2 = 12 months after CVD treatment; EVTA = endovenous thermal ablation.

a Patients lost to follow-up (n = 27) were not included.

From T0 to T2, the rate of patients with three items score ≥4 and ASEX total score ≥19 was significantly reduced (three items score ≥4: 95.7 % vs 31.5 %, OR 0.02, CI95 % 0.005–0.06, p-value= <0.001; ASEX total score ≥19: 100 % vs 20 %, p-value= <0.001) (Table 4).

**Table 4:** Improvement quantification of ASEX scores in the subgroup affected by SD, 12 months from CVD treatment (T2).

CVD +SD *(n = 95*, *14.6 %)*^a^					
	T0	T2	Odds Ratio	CI 95 %	p-value *(<0.05)*
ASEX total score ≥ 19	95 (100 %)	19 (20 %)	NA	NA	<0.001
One item with a score of ≥ 5	18 (18.9 %)	8 (8.4 %)	0.39	[0.14; 1.02]	0.055
Three items with a score of ≥ 4	91 (95.7 %)	30 (31.5 %)	0.02	[0.005; 0.06]	<0.001

*Footnotes:* CVD = chronic venous disease; SD = sexual dysfunction; T0 = time of CVD diagnosis; T2 = 12 months after CVD treatment; NA = not applicable.

a Patients lost to follow-up (n = 27) were not included.

**Fig. 1:**
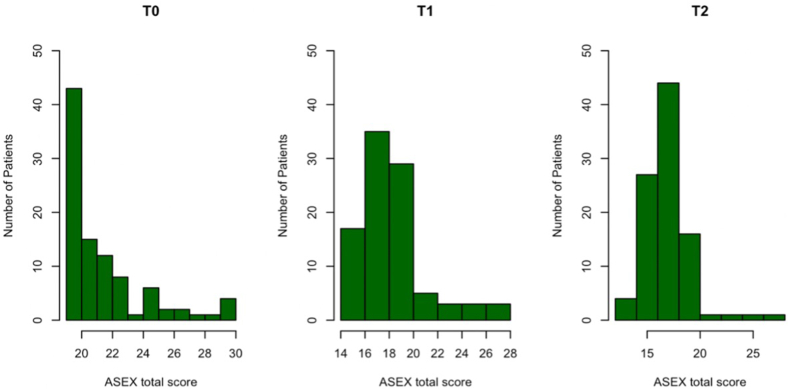
Distribution of ASEX total score before (T0) and after (T1, T2) CVD treatment in the subgroup affected by SD. Footnotes. CVD = chronic venous disease; SD = sexual dysfunction; T0 = time of CVD diagnosis; T1 = 6 months after CVD treatment; T2 = 12 months after CVD treatment.

Distribution of ASEX total score before (T0) and after (T1, T2) CVD treatment in the subgroup affected by SD is shown in Fig. 1.

**Fig. 2:**
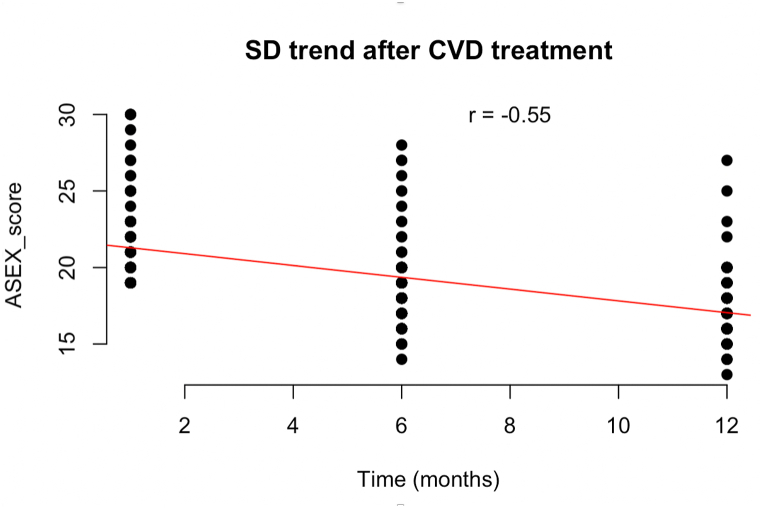
Pearson's correlation between ASEX score and time after CVD treatment in the subgroup affected by SD [ r = −0.55, CI95 % (−0.62; −0.46), p-value= <0.001] Footnotes. CVD = chronic venous disease; SD = sexual dysfunction; r = correlation coefficient.

Pearson's correlation study showed a significant negative association between ASEX total score and time from CVD treatment (r = −0.55, CI95 % −0.62; −0.46, p-value= <0.001) (Fig. 2).

## 4 Discussion

CVD clinical manifestations can determine a series of social problems and can also hinder daily activities, that can ultimately worsen health related QoL of affected patients [13,14]. Several common health conditions and their treatments are associated with SD [15], but this is the first study evaluating SD in CVD patients.

Sexual function is an innate and natural dimension of human health, is strictly related to life experiences and interpersonal relationships, and several chronic diseases can affect it considerably. Sexual health is also related to social expectations, social psychological growth, and is directly related to the structuring of social and sexual identity. All these factors influence patients’ perceived QoL, which is a significant measure complementing clinical assessment in the view of precision medicine that is related to holistic and tailored patient care [[16], [17], [18], [19]].

Moreover, SD is also able to undermine the relationship of affected patients with their partners, and this may also lead to loss of social contact, isolation, and feeling of loneliness, all of which may influence negatively the other underlying diseases [16].

In our study 122 CVD patients (18.8 %), (42 males and 80 females) resulted affected from SD. Patients with more severe forms of CVD (C3–C6) were more likely to have SD, confirming the role of CVD in the onset of SD. Furthermore, CVD patients with SD after 12 months of treatment showed a significant improvement of SD, regardless the specific type or combination of management (VADs, compression therapy, open surgery, endovascular surgery). Generally, it is clearly established that there is a close correlation between developments in SD and the course of several related chronic diseases. In fact, if an improvement occurs in one often some improvements occur in the other [20].

CVD affects the well-being of the patients, both at the physical and the psychological levels [21], impacting also CVD patients ‘perception of their body image. In fact, clinical signs such as varicose veins, leg swelling, skin changes, and ulceration can alter the shape, the color and the consistency of the skin and all the normal features of the lower limbs determining negative perception of the body image. In this study, patients with C3–C6 clinical classes (more severe clinical stages) were more likely to have SD respect to C2 patients (mild clinical stage). Moreover, the concerns of people, particularly women, about how others may evaluate their body can significantly affect their sexual function [22]. Accordingly, in our study 65.5 % of CVD patients with SD were women, thus gender diversity is an important factor in health issues assessment [23]. Moreover, the negative emotional impact of a patient suffering from a chronic disease may be is exacerbated by its impact on self-esteem and self-worth, if the chronic disease affects body-image, and all this may lead to the onset of SD [20].

These results are in line with other studies according to which women's body image is a complex dimension and has been conceptualized as body part satisfaction (i.e., satisfaction with stomach, arms, thighs), related also with body size or weight, and with the comfort with one's body in front of a partner or [24]. These feelings or perceptions about one's body are often influenced by sociocultural and intrapersonal issue and can impact sexual health. Changes in body image perception are mostly evident among women affected by health problems [25].

In this case the chronic disease may negatively modify the patient's affective and emotional experience [20], especially, as in the case of CVD, if the length of the disease matches the patient's life [1].

Moreover, vascular diseases in general may cause depressive symptoms that may determine compromission of sexual desire, and of the sexual performance. Importantly, sex health may be affected also by the distress with one's sexual difficulties and the concerns about a partner's perception of those sexual difficulties. In fact, these issues may be significantly associated with decreased one's body esteem [26].

The underlying pathophysiology related to depression can affect the ability to relax the smooth muscles of erectile tissue, reducing also sexual psychogenic stimuli, resulting in impaired sexual response cycle [27].

On one side sexual health is strictly related to social determinants of health (SDHs) [28], and on the other side SDHs are also related to CVD [14]. Thus, in the presence of chronic illness, such as CVD, it is important to evaluate to evaluate all the factors that can impair social function in affected patients, as social issues can interfere with clinical course of vascular disease and their response to treatment [14].

In this study, comorbidities such as hypertension, dyslipidemia, and CAD were more frequent in CVD +SD group. In particular, in the current literature the impact of CAD has been showed especially for men, but the association is not fully established yet [29]; hypertension and related treatment seem to exert a detrimental impact on sexual function while the role of dyslipidemia and related statin treatment is not yet clear [30].

The limitations of this study are mainly represented by the cross-sectional design that does not allow to document causal relationship and by the tool used to study SD, the questionnaire, that may determine social desirability bias [31].

## 5 Conclusions

In conclusion, CVD patients may experience SD, especially in female sex, in more advanced disease stages and with some comorbidities such as hypertension, dyslipidemia, and CHD. SD in CVD patients appears to improve after adequate CVD treatment. Further research is needed in order to clarify this association. Physicians may consider assessing the relation between CVD and body image when patients present with sexual problems, as distress and distraction over the appearance of their bodies may be substantially contributing to their overall sexual satisfaction independent of their sexual function.

## Declarations

### 5.1 Ethics approval and consent to participate

The study was approved by the Institutional Review Board of Interuniversity Center of Phlebolymphology (CIFL) International Research and Educational Program in Clinical and Experimental Biotechnology (Approval number: ER.ALL.2018.64A.). All patients gave written informed consent.

### Consent for publication

Not applicable.

### Data availability statement

Data associated with this study has not been deposited into a publicly available repository as all data generated or analyzed during this study are included in this article.

### Funding

This research received no external funding.

## CRediT authorship contribution statement

**Davide Costa:** Writing – review & editing, Writing – original draft, Methodology, Investigation, Conceptualization. **Nicola Ielapi:** Writing – original draft, Investigation. **Umberto Marcello Bracale:** Writing – original draft, Investigation. **Antonio Peluso:** Writing – original draft, Investigation. **Roberto Minici:** Writing – original draft, Investigation. **Michele Andreucci:** Writing – original draft, Investigation. **Raffaele Serra:** Writing – review & editing, Writing – original draft, Methodology, Investigation, Conceptualization.

## Declaration of competing interest

The authors declare that they have no known competing financial interests or personal relationships that could have appeared to influence the work reported in this paper.

## List of abbreviations

ASEX: Arizona Sexual Experience
BMI: Body Mass Index
CAD: Coronary Artery Disease
CEAP: Clinical, Etiological
Anatomical: and Pathophysiological classification
CKD: Chronic Kidney Failure, COPD Chronic Obstructive Pulmonary Disease
CVD: Chronic Venous Disease, CVLUs Chronic Venous Leg Ulcers
CVU: Chronic Venous Ulceration, DAP Diastolic Arterial Blood Pressure
DOAC: Direct Oral Anticoagulant, EVTA Endovenous Thermal Ablation
QoL: Quality of Life
SAP: Systolic Arterial Blood Pressure
SD: Sexual Dysfunction
abrSDHs: Social Determinants of Health
VADs: Veno-Active Drugs

## References

[1] Serra R., Grande R., Butrico L., Fugetto F., de Franciscis S. (2016). Epidemiology, diagnosis and treatment of chronic venous disease: a systematic review. Chirurgia.

[2] Radak D.J. (2013). Quality of life in chronic venous disease patients measured by short Chronic Venous Disease Quality of Life Questionnaire (CIVIQ-14) in Serbia. J. Vasc. Surg..

[3] Ortega-Santana F. (2014). The influence of the CIVIQ dimensions on quality of life of patients with primary superficial venous incompetence. Eur. J. Vasc. Endovasc. Surg. : the official journal of the European Society for Vascular Surgery.

[4] Lozano Sánchez F.S. (2013). Quality of life in patients with chronic venous disease: influence of the socio-demographical and clinical factors. Int. Angiol. : a journal of the International Union of Angiology.

[5] Launois R. (2015). Health-related quality-of-life scales specific for chronic venous disorders of the lower limbs. Journal of vascular surgery. Venous and lymphatic disorders.

[6] Nicolaides A., Kakkos S., Baekgaard N. (2018). Management of chronic venous disorders of the lower limbs. Guidelines According to Scientific Evidence. Part I. Int. Angiol. : a journal of the International Union of Angiology.

[7] Bootun R., Burrows M., Chowdhury M.M., Stather P.W., Al-Jundi W. (2023). The risk of harm whilst waiting for varicose veins procedure. Phlebology.

[8] Boyacıoğlu N.E. (2023). E. Sexuality, quality of life and psychological well-being in older adults: a correlational study. Eur. J. Obstet. Gynecol. Reprod. Biol. X.

[9] Terentes-Printzios D., Ioakeimidis N., Rokkas K., Vlachopoulos C. (2022). Interactions between erectile dysfunction, cardiovascular disease and cardiovascular drugs. Nat. Rev. Cardiol..

[10] Lurie F. (2020). The 2020 update of the CEAP classification system and reporting standards. Journal of vascular surgery. Venous and lymphatic disorders.

[11] McGahuey C.A. (2000). The Arizona sexual experience scale (ASEX): reliability and validity. J. Sex Marital Ther..

[12] Elnazer H.Y., Baldwin D.S. (2020). Structured review of the use of the Arizona sexual experiences scale in clinical settings. Hum. Psychopharmacol..

[13] Silva W.T. (2020). Differences in health-related quality of life in patients with mild and severe chronic venous insufficiency: a systematic review and meta-analysis. J. Vasc. Nurs..

[14] Costa D. (2023). Social determinants of health and vascular diseases: a systematic review and call for action. Soc. Sci..

[15] Flynn K.E. (2016). Sexual satisfaction and the importance of sexual health to quality of life throughout the life course of U.S. Adults. J. Sex. Med..

[16] Tański W., Dudek K., Tomasiewicz A., Świątoniowska-Lonc N. (2022). Sexual dysfunction and quality of life in patients with rheumatoid arthritis. Int. J. Environ. Res. Publ. Health.

[17] Deutsch A.R., Hoffman L., Wilcox B.L. (2014). Sexual self-concept: testing a hypothetical model for men and women. J. Sex. Res..

[18] Potki R., Ziaei T., Faramarzi M., Moosazadeh M., Shahhosseini Z. (2017). Bio-psycho-social factors affecting sexual self-concept: a systematic review. Electron. Physician.

[19] Ielapi N. (2020). Precision medicine and precision nursing: the Era of biomarkers and precision health. Int. J. Gen. Med..

[20] Colson M.H. (2016). Sexual dysfunction and chronic illness. Part 1. Epidemiology, impact and significance. Sexologies.

[21] Branisteanu D.E., Feodor T., Baila S., Mitea I.A., Vittos O. (2019). Impact of chronic venous disease on quality of life: results of vein alarm study. Exp. Ther. Med..

[22] Afshari P., Houshyar Z., Javadifar N., Pourmotahari F., Jorfi M. (2016). The relationship between body image and sexual function in middle-aged women. Electron. Physician.

[23] Costa D. (2023). Diversity and health: two sides of the same coin. Italian Sociological Review.

[24] Schick V.R., Calabrese S.K., Rima B.N., Zucker A.N. (2010). Genital appearance dissatisfaction: implications for women's genital image self-consciousness, sexual esteem, sexual satisfaction, and sexual risk. Psychol. Women Q..

[25] Lowenstein L. (2009). Sexual function is related to body image perception in women with pelvic organ prolapse. J. Sex. Med..

[26] Pujols Y., Seal B.N., Meston C.M. (2010). The association between sexual satisfaction and body image in women. J. Sex. Med..

[27] Nascimento E.R. (2013). Sexual dysfunction and cardiovascular diseases: a systematic review of prevalence. Clinics.

[28] Rao T.S., Gopalakrishnan R., Kuruvilla A., Jacob K.S. (2012). Social determinants of sexual health. Indian J. Psychiatr..

[29] Steptoe A., Jackson S.E., Wardle J. (2016). Sexual activity and concerns in people with coronary heart disease from a population-based study. Heart (British Cardiac Society).

[30] Imprialos K.P. (2018). Sexual dysfunction, cardiovascular risk and effects of pharmacotherapy. Curr. Vasc. Pharmacol..

[31] Krumpal I. (2013). Determinants of social desirability bias in sensitive surveys: a literature review. Qual. Quantity.

